# Analysis of the CaMKIIα and β splice-variant distribution among brain regions reveals isoform-specific differences in holoenzyme formation

**DOI:** 10.1038/s41598-018-23779-4

**Published:** 2018-04-03

**Authors:** Sarah G. Cook, Ashley M. Bourke, Heather O’Leary, Vincent Zaegel, Erika Lasda, Janna Mize-Berge, Nidia Quillinan, Chandra L. Tucker, Steven J. Coultrap, Paco S. Herson, K. Ulrich Bayer

**Affiliations:** 1 Department of Pharmacology, University of Colorado Anschutz Medical Campus, Aurora, Mail Stop 8303, RC1-North, 12800 East 19th Ave, Aurora, CO 80045 USA; 2 Department of Anesthesiology, University of Colorado Anschutz Medical Campus, Aurora, Mail Stop 8321, RC1-South, 12800 East 19th Ave, Aurora, CO 80045 USA; 3 Present Address: Department of Chemistry and Biochemistry, University of Colorado, Boulder, USA

## Abstract

Four CaMKII isoforms are encoded by distinct genes, and alternative splicing within the variable linker-region generates additional diversity. The α and β isoforms are largely brain-specific, where they mediate synaptic functions underlying learning, memory and cognition. Here, we determined the α and β splice-variant distribution among different mouse brain regions. Surprisingly, the nuclear variant αB was detected in all regions, and even dominated in hypothalamus and brain stem. For CaMKIIβ, the full-length variant dominated in most regions (with higher amounts of minor variants again seen in hypothalamus and brain stem). The mammalian but not fish CaMKIIβ gene lacks exon v3_N_ that encodes the nuclear localization signal in α_B_, but contains three exons not found in the CaMKIIα gene (exons v1, v4, v5). While skipping of exons v1 and/or v5 generated the minor splice-variants β’, βe and βe’, essentially all transcripts contained exon v4. However, we instead detected another minor splice-variant (now termed βH), which lacks part of the hub domain that mediates formation of CaMKII holoenzymes. Surprisingly, in an optogenetic cellular assay of protein interactions, CaMKIIβH was impaired for binding to the β hub domain, but still bound CaMKIIα. This provides the first indication for isoform-specific differences in holoenzyme formation.

## Introduction

The Ca^2+^/calmodulin-dependent protein kinase II (CaMKII) constitutes a family of closely related protein kinase isoforms (α, β, γ, and δ) that are encoded by four distinct genes (for review see^1,2^). CaMKIIγ and δ are ubiquitously expressed, whereas CaMKIIα and β expression is largely restricted to the brain^3,4^, with the β isoform additionally expressed in pancreas and skeletal muscle^4–6^. CaMKII expression is extremely high in the brain, where CaMKII mediates forms of bi-directional synaptic plasticity that underlie learning, memory and cognition^7–12^. Indeed, the CaMKIIα knockout was the first described genetically engineered mouse model with a behavioral phenotype in learning and memory^13^.

**Figure 1:**
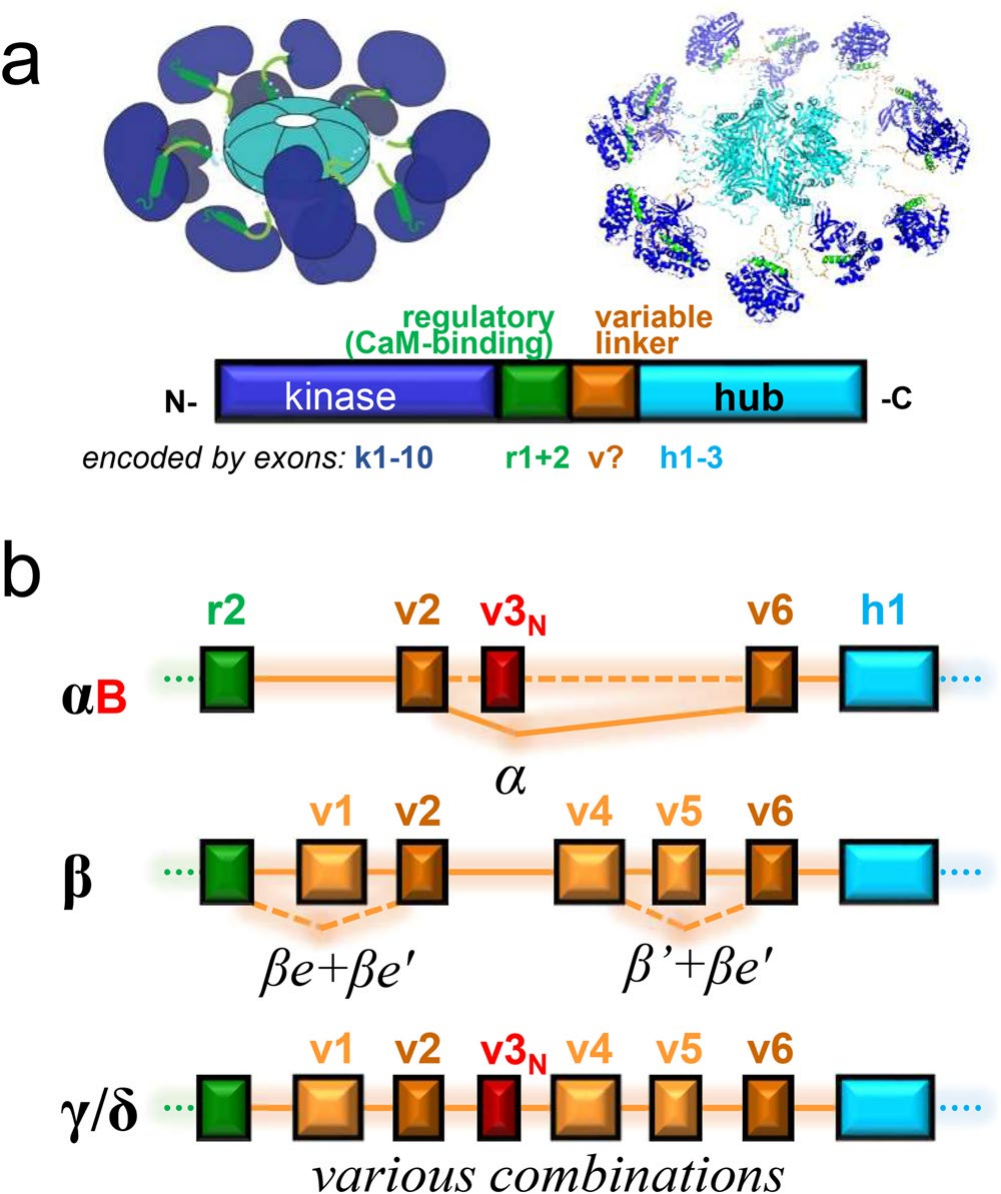
Structure of CaMKII isoforms and splice-variants. (**a**) The CaMKII holoenzyme structure (upper panels) and protein domain sequence (lower panel). An N-terminal kinase domain (blue; encoded by 10 exons) is followed by the Ca^2+^/CaM-binding regulatory domain (green; encoded by two exons), the variable linker domain (orange; subject to alternative splicing), and the C-terminal hub domain (aqua; encoded by three exons) that mediates holoenzyme formation. (**b**) The exons encoding the variable domain of the four mammalian CaMKII isoforms in comparison. Exclusion of specific exons in specific splice variants of the α and β isoforms are indicated. Note that exons v2 and v6 (dark orange) appears to be included in almost all splice variants of all CaMKII isoforms; inclusion of exon v3_N_ (red) generates a functional nuclear localization signal.

Each CaMKII isoform contains an N-terminal kinase domain, followed by a Ca^2+^/CaM-binding autoinhibitory regulatory domain, a variable linker-region, and a C-terminal hub domain (also termed association domain) that mediates the formation of 12meric holoenzymes (see Fig. 1a)^1,2,14^. The variable linker-region is subject to alternative splicing in all four CaMKII isoforms, which in turn can affect regulation and subcellular targeting^1,2,15–18^. This variable linker-region is where CaMKIIα and β differ most, and it mediates the high-affinity F-actin binding that is specifically seen for CaMKIIβ but not α (or the β splice-variants βe and βe’; see Fig., 1b); consequently, this difference is thought to be responsible for distinct functions of these two brain-specific isoforms^19–27^.

The CaMKIIα variable linker-region is encoded by only two exons, v2 and v6, and these two exons appear to be present in almost all splice-variants of all CaMKII isoforms (see Fig. 1b). A minor splice-variant, αB, additionally contains the alternative exon v3_N_ that generates a functional nuclear localization signal (indicated in red in Fig. 1b); a homologous sequence has also been described in splice-variants of CaMKIIγ and δ^16,17,28^, but not β.

The CaMKIIβ variable linker-region is encoded by five exons (v1, v2, v4–v6), with homologous exons found also in CaMKIIγ and δ (Fig. 1b). Additionally, the CaMKIIβ gene contains three unique exons (termed v7a–c) not found in any other isoform; two of these exons are included in the β3 variant that is found in pancreatic β-cells^5^, and all three are included in the βM variant that is the major β-variant in skeletal muscle^4,6^. For CaMKIIβ, splice-variants lacking exon v1 and/or v5 have been described (βe, βe’, and β’; see Fig., 1b), with βe dominating during early development and abolishing F-actin binding^16,21^. However, in contrast to the γ and δ isoforms, no rodent brain CaMKIIβ variants have been described to either lack exon v4 or to include exon v3_N_.

All CaMKII isoforms can form holoenzymes with each other, with no known isoform-specific preference^6,29,30^. Indeed, all isoforms share extensive homology^2,31^. Thus, arguably, splice-variants can differ more from each other than isoforms. This is certainly the case for splice-variants that generate nuclear targeting signals (such as αB)^16^ or that abolish high-affinity F-actin binding (such as βe and βe’)^21^. However, while the CaMKII isoform distribution has been examined among different tissues (by Northern blot)^3,4^ and brain regions (largely by *in situ* hybridization)^4,32–35^, the distribution of the different splice-variants has not been studied systematically.

Here, we identify the distribution of CaMKIIα and β splice-variants among different mouse brain regions. Surprisingly, we found that expression of the nuclear αB variant is much more widespread than anticipated and that it is even the dominant α variant in some regions, such as the hypothalamus. For CaMKIIβ, the full-length β variant dominated in most brain regions. While β’, βe, and βe’ were also widely detectable, β variants lacking exon v4 (or including exon v3_N_) were not. However, we did identify new unusual minor splice-variants of CaMKIIβ that lack part of hub domain exon h2 (now termed βH and βeH). Notably, the βH hub domain showed impaired binding to the full-length hub domain of CaMKIIβ, but surprisingly still interacted with CaMKIIα. This provides the first indication for isoform-specific differences in holoenzyme formation.

## Results

### Expression of CaMKIIα versus α_B_ transcripts among different mouse brain regions

**Figure 2:**
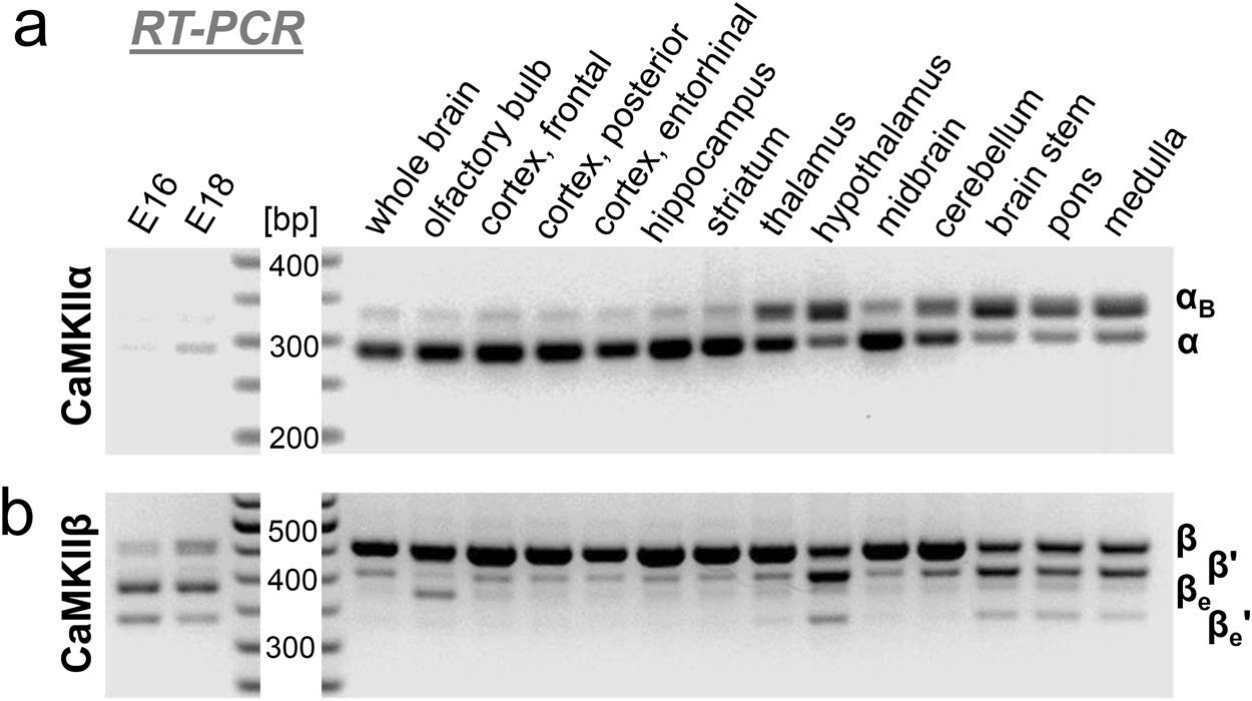
Distribution of CaMKIIα and β splice-variant transcripts among mouse brain regions, detected by RT-PCR with primers flanking the variable linker-region of α or β isoforms, respectively (with primers directed against α exons r1/2 and h3 or β exons r2 and h1). Additionally, expression in late-stage mouse embryos on embryonal day E16 or E18 was analyzed (and the data shown are from the same exposure of the same gel shown for the brain regions). (**a**) For CaMKIIα, expression was detected in all regions of adult brain, with only minimal expression detected in late stage embryos. Surprisingly, expression of the nuclear αB variant was detected in all brain regions; in hypothalamus, pons, and medulla, αB was even the dominant variant. (**b**) For CaMKIIβ, expression of the full-length β variant dominated in most adult brain regions, while the βe variant dominated in embryos. Some expression of β, β’, βe, and βe’ was detected in all brain regions, with β’ typically being the second most dominant variant after the full-length β.

CaMKIIα and α_B_ transcripts were amplified by RT-PCR, using primers flanking the variable region^16^ (see Fig. 1b). As expected, CaMKIIα was by far the dominant α variant in whole brain from adult mice, and only minute expression of any α-related transcript was detected in mouse embryos (Fig. 2a). CaMKIIα was also the dominant α variant in many individual brain regions; in particular, this included the olfactory bulb, neocortex, hippocampus, and striatum. However, surprisingly, in several brain regions, transcripts for the nuclear αB variant predominated instead; this included the brain stem (including both pons and medulla) and the hypothalamus (Fig. 2a). In thalamus and cerebellum, αB transcripts were also more prominent, even though the α transcripts still dominated. Additionally, some level of αB expression was detected in any brain region examined. Overall, it appeared that the relative expression of αB transcripts was higher in brain regions that showed lower overall expression of the CaMKIIα isoform.

### Expression of CaMKIIβ splice-variant transcripts during development and among different brain regions

CaMKIIβ splice-variant transcripts were amplified by RT-PCR, using primers flanking the variable region (see Fig. 1b). As shown previously^21^, four distinct β variants were amplified with these primers and resolved by gel electrophoresis: CaMKIIβ, β’, βe, and βe’ (Fig. 2b). As expected, CaMKIIβ predominated in whole brain from mature mice, while βe predominated in embryos at ages E16 and E18. In all brain regions tested, all four splice-variants were detectable. The full-length CaMKIIβ transcripts predominated in most brain regions, but approximately equal β and β’ expression was seen in hypothalamus and brainstem (i.e. in pons and in the medulla; Fig. 2b). In other regions of the mature mouse brain, β’ was the second most abundant splice-variant, even though its expression was only a fraction compared to full-length CaMKIIβ (Fig. 2b). The only exception was the olfactory bulb, where βe was more abundant than β’ (but both were still much less abundant than β).

### Western analysis of CaMKIIα and β splice-variant expression

**Figure 3:**
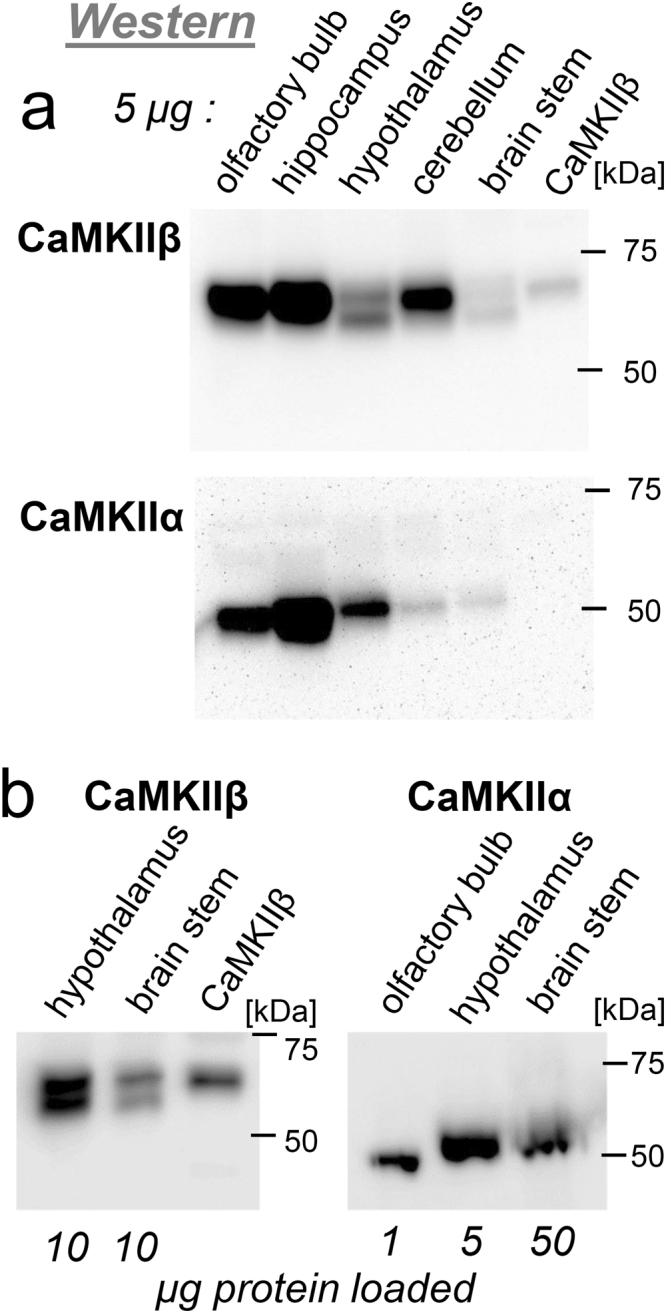
Western analysis of CaMKIIα and β protein expression. (**a**) Total protein from five brain regions (5 µg each) and CaMKIIβ expressed in HEK293 cells were separated on a 10% polyacrylamide SDS gel and subjected to Western analysis with specific antibodies against CaMKIIβ and α. Hypothalamus and brain stem contains an additional shorter CaMKIIβ variant; the CaMKIIα in these brain regions appears to run slightly higher (consistent with the 1 kDa heavier αB). (**b**) Western analysis of larger protein amounts (10 µg) enabled better visualization of the CaMKIIβ double band in brain stem; adjusted protein amounts (1–50 µg, as indicated) and longer run on a 12% polyacrylamide SDS gel enabled better distinction of a longer CaMKIIα band in hypothalamus and brain stem.

The PCR analysis above indicated that the CaMKIIα and β variants dominate in most brain regions, except for brain stem and hypothalamus, which instead showed higher or equal expression of CaMKIIαB or β’ (see Fig. 2). Thus, we decided to test brain stem and hypothalamus for expression of splice-variants at the protein level by Western blot; additional brain region tested for comparison included olfactory bulb, hippocampus, and cerebellum (Fig. 3a). Indeed, in hypothalamus, a shorter CaMKIIβ variant was predominant (Fig. 3a), as predicted from the RT-PCR analysis. Two CaMKIIβ bands were also detected in brain stem (Fig. 3a), which became more obvious when more protein was loaded (Fig. 3b). For CaMKIIα, a fair amount of protein was detected in hypothalamus, and much less in cerebellum and brain stem (Fig. 3a). RT-PCR predicted αB to be predominant in hypothalamus and brain stem, however, the 1.25 kDa difference between α and αB did not resolve on the 10% polyacrylamide SDS gel, although the CaMKIIα variant in hypothalamus appeared to run slightly higher than in olfactory bulb and hippocampus (Fig. 3a); this difference became more apparent when a 12% polyacrylamide SDS gel was run longer (Fig. 3b; note also adjusted protein amounts).

### Increased nuclear localization of CaMKIIα in hypothalamus compared to cortex

**Figure 4:**
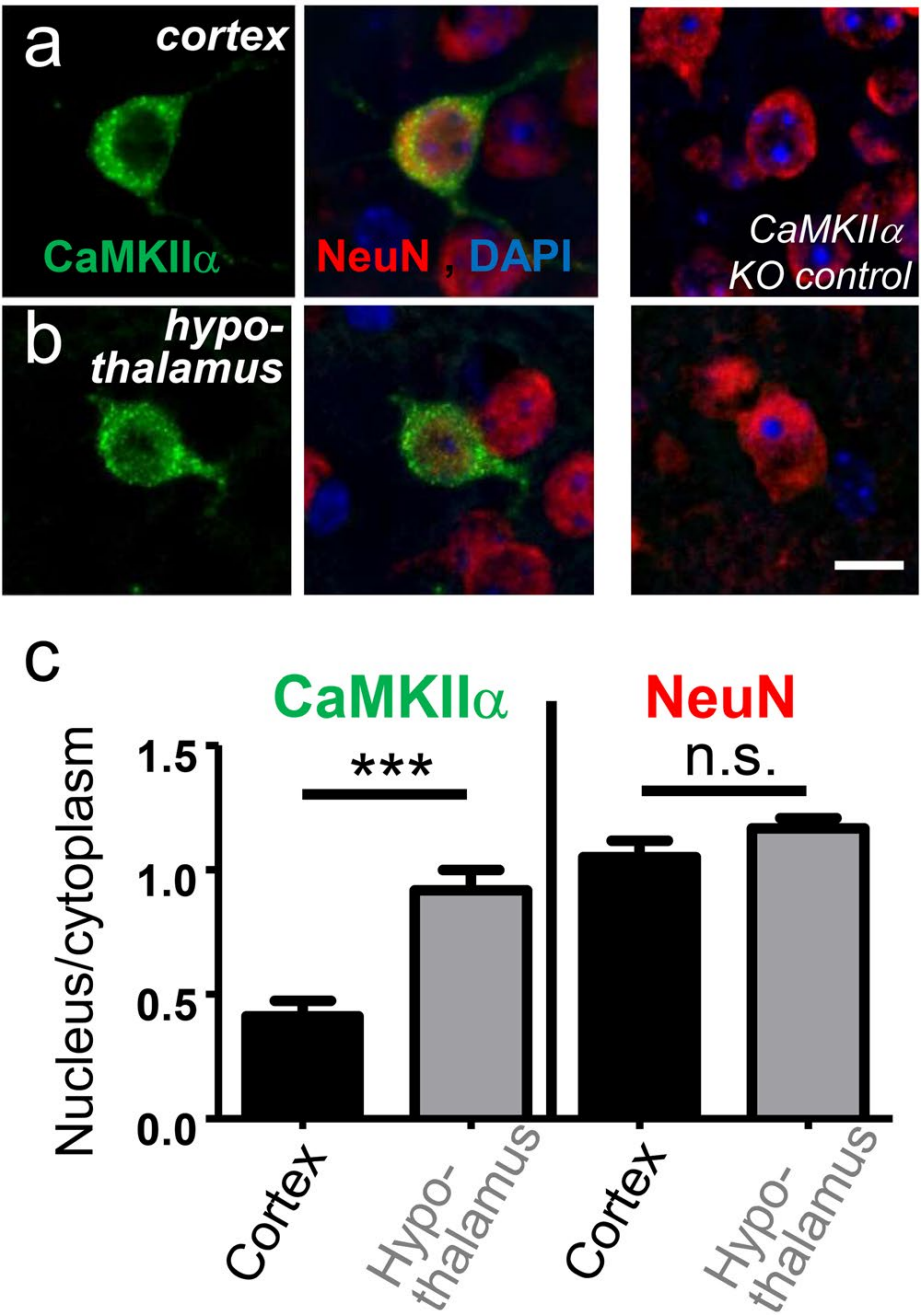
Immunohistochemistry of CaMKIIα in mouse brain, specifically in (**a**) cortex and (**b**) hypothalamus. Specificity was verified by lack of staining in tissue from CaMKIIα ko mice. Neurons were stained with an antibody against NeuN; nuclei were stained with DAPI. (**c**) Quantification showed significantly higher ratio of nuclear over cytoplasmic CaMKIIα localization in hypothalamus compared to cortex, as expected based on the higher ratio of αB variant expression. The relative nuclear localization of the NeuN stain was undistinguishable between the brain regions. ***p < 0.0001; ns: p > 0.05; n = 11 or 10 cells (cortex or hypothalamus, respectively). Scale bars, 10 µm.

In order to determine if nuclear localization of CaMKIIα is increase in hypothalamus (the brain region with the highest relative expression of the nuclear αB variant), we performed immunohistochemistry on paraffin-embedded sagittal slices of mouse brain (Fig. 4). Specificity of the antibody was verified by lack of staining in slices from CaMKIIα knock out mice (Fig. 4a,b); nuclei were identified by DAPI staining and neuron were identified by staining for NeuN (Fig. 4a,b). In cortical neurons, CaMKIIα appeared to be largely cytoplasmic (Fig. 4a), while cortical neurons appeared to have additional more extensive nuclear CaMKIIα staining (Fig. 4b). Indeed, quantification showed significantly more extensive nuclear localization in hypothalamus compared to cortex (Fig. 4c). Notably, the degree of nuclear CaMKIIα appeared quite variable among individual neurons in the hypothalamus. Nonetheless, as expected, the increased expression of the αB variant in the hypothalamus was positively correlated with overall increased nuclear localization.

### The CaMKIIβ gene of fish but not mammals contains an exon v3_N_ homologue

The CaMKIIα, γ and δ genes contain a variable region exon (v3_N_) that generates a functional nuclear localization signal (see Fig. 1b), specifically in the splice-variants αB^16^, γA^28^, and δB^17^. In order to determine if the CaMKIIβ gene may contain an exon homologous to v3_N_, we analyzed the genomic regions of mouse, rat and human CaMKIIβ between exons v2 and v4. We searched the genomic regions first for sequences homologous to the exon v3_N_ of CaMKIIα, then for sequences that could code for Lys-Arg-Lys (i.e. AA[A/G] CGX AA[A/G]), the part of the nuclear localization signal encoded by exon v3_N_. No such exon v3_N_ homologues were found in any available mammalian CaMKIIβ gene sequences. However, CaMKIIβ sequences that include exon v3_N_ were found for several species of bony fish and sharks (including zebrafish and whale shark, GenBank accession numbers XP_021331923 and XP_020367006). Thus, while exon v3_N_ appears to be absent from mammalian CaMKIIβ, it is found in some vertebrate species.

### CaMKIIβ transcripts lacking exon v4 are not detectable in rat brain by RT-PCR

**Figure 5:**
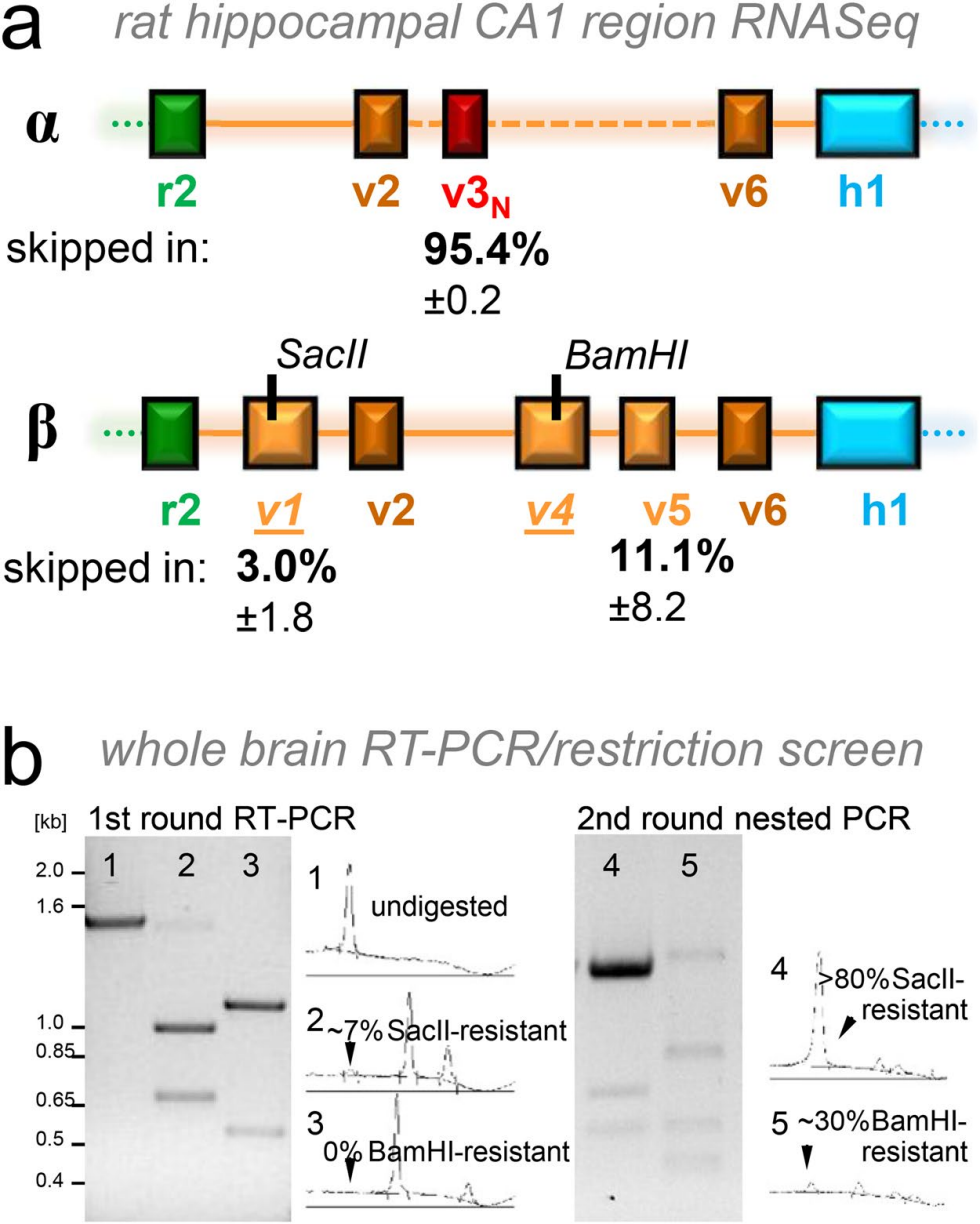
The CaMKIIβ splice-variants in mature rat brain essentially all contain exon v4. (**a**) RNA Seq indicated that most hippocampal CaMKIIα transcripts lack exon v3_N_ and that some hippocampal CaMKIIβ transcripts lack exon v1 or v5 sequences (with percentages indicated), but all contained exons v2, v4, and v6. A BamHI restriction site in β exon v4 can be utilized for a PCR/restriction strategy to identify transcripts lacking the exon v4 sequence: After a first round PCR, restriction digest with BamHI eliminates all templates that contain exon v4 for amplification in a second round of PCR with nested primers. As a positive control, a parallel PCR/restriction approach with SacII should identify the known variant βe that lacks exon v1. (**b**) A first round RT-PCR generated a band of the expected size (lane 1); ~7% of this band was resistant to digestion with SacII, indicating lack of exon v1 (lane 2); resistance to BamHI was not detected, indicating no lack of exon v4 (lane 3). (**c**) A second round of PCR after SacII digest yielded PCR products that were largely resistant to SacII (lane 4); a second round of PCR after BamHI digest yielded some BamHI resistant PCR product (lane 5), however, cloning and sequencing of these products did not reveal any products that lacked exon v4 (some had mutations in the BamHI site, while others were non-specific products unrelated to CaMKIIβ.

Our RT-PCR analysis showed β splice-variants lacking exon v1 and/or v5, but did not indicate variants lacking v4. As lack of exon v4 has been described for splice-variants of both CaMKIIγ and δ^31^, we attempted detection of CaMKIIβ transcripts in rat brain that specifically lack v4, using two different strategies. First, RNA sequencing (RNA Seq) indicated that of all CaMKIIβ transcripts, ~3% lacked exon v1 and ~11% lacked exon v5, while no transcripts lacking exons v2, v4, or v6 were detected in the hippocampal CA1 region (Fig. 5a). (The same analysis indicated that ~4.6% of CaMKIIα contain the NLS encoded by exon v3_N_, a finding consistent with ratio of transcripts that were detected by RT-PCR). Then, in order to test if exon v4 might be lacking in transcripts from other brain regions, we utilized a nested RT-PCR/restriction strategy on whole brain RNA: After a first round of amplification that results in a ~1.6 kb product, we digested the PCR product with BamHI, a restriction enzyme that cuts only within exon v4 of the amplified CaMKIIβ region (Fig. 5a). In a second round of nested PCR designed to result in a ~1.2 kb product, this BamHI restriction digest should prevent further amplification of any PCR product that contains the BamHI restriction site located in exon v4. As a positive control, we used restriction digest with SacII, and enzyme that cuts in exon v1 (Fig. 5a); this exon is lacking in the CaMKIIβ splice-variant βe (a splice-variant that is even more rare than β’ in the adult brain; see Fig. 2b). After the first round of RT-PCR, ~7% of the PCR product was resistant to digest by SacII, while no BamHI resistant products were detectable (Fig. 5b). After the second round of nested PCR after SacII digest, more than 80% of the product was SacII resistant. By contrast, a second round of nested PCR after BamHI digest yielded much less product and only ~30% of this second round product was BamHI resistant (Fig. 5c). Both the SacII- and the BamHI-resistant second round products were gel-purified and cloned into a bacterial vector for further analysis. Of 18 clones obtained from the BamHI restriction strategy, six clones were not actually BamHI resistant and three contained sequences that were unrelated to CaMKII. Of the remaining nine clones, seven clones contained point mutations within the BamHI site, but still contained exon v4. The remaining two clones contained deletions of the BamHI site, but these deletions did not correspond to any exon/intron borders and both resulted in a reading frame shift. Thus, while our strategy successfully and efficiently amplified the minor splice-variant βe (lacking exon v1 that contains the SacII restriction site), it did not detect any bona fide CaMKIIβ transcripts that lack exon v4.

### CaMKII splice-variants with a deletion within the hub domain

**Figure 6:**
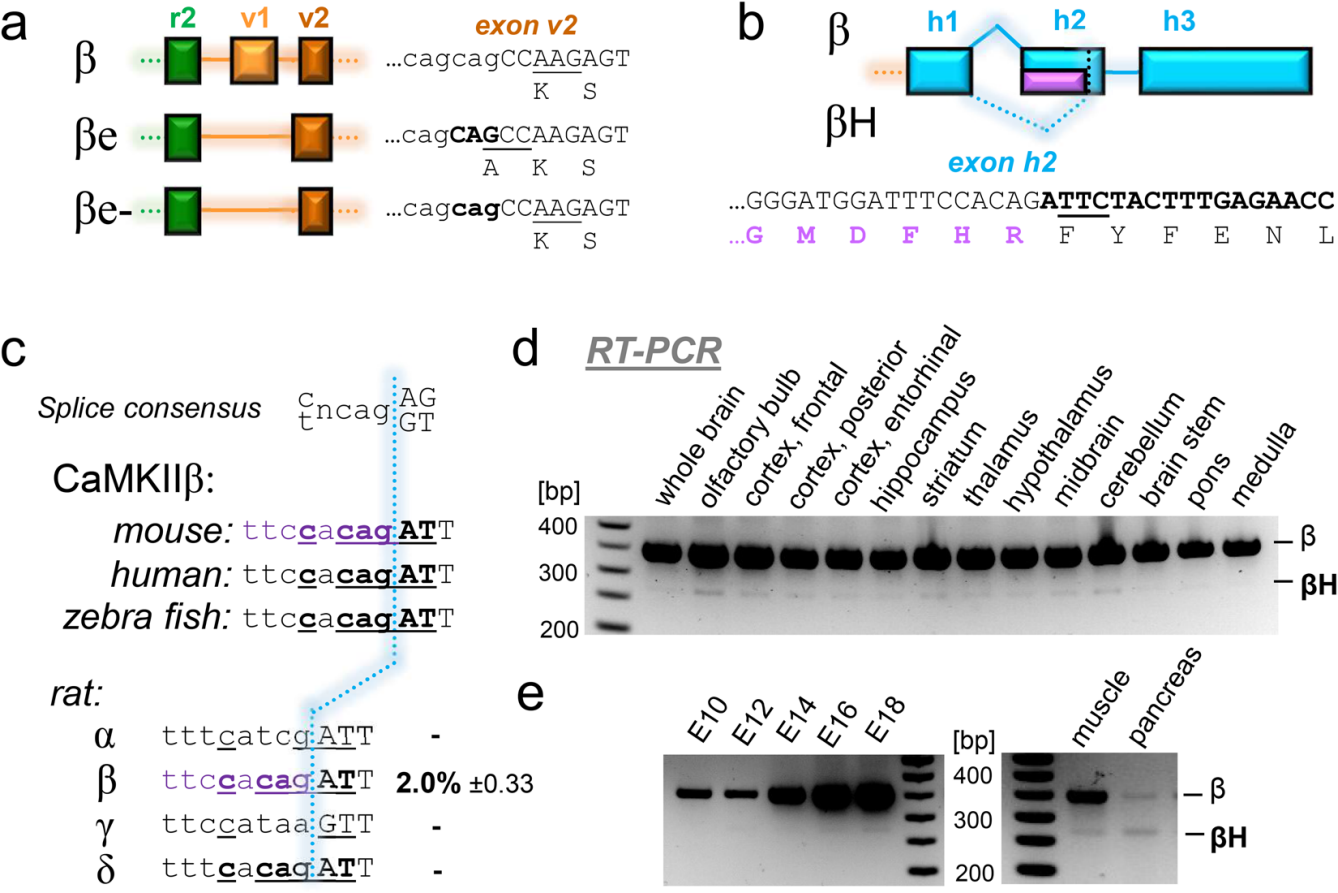
Unexpected novel CaMKII splice-variants that lack part of hub domain exon h2. (**a**) CaMKIIβ exon v2 contains an additional splice acceptor, that appears to be utilized only when exon v1 is skipped, such as in the originally described βe variant; here, sequence analysis additionally showed a βe variant without the one amino acid insertion created by the additional splice acceptor. (**b**) Partial skipping of hub domain exon h2 generates the novel CaMKIIβ splice variant βH. This is enabled by an additional splice acceptor site within exon h2. (**c**) The CaMKIIβ exon h2 internal splice site has the consensus sequence for splice acceptor sites that is conserved among species. The same consensus site is found also in CaMKIIδ, but not α or γ. RNA Seq of the rat hippocampal CA1 region detected alternative h2 splicing only in the β isoform (with percentage of transcripts indicated), but in none of the other isoforms. (**d**) The novel CaMKIIβH is a minor splice variant in all mouse brain regions, as shown by RT-PCR with primers flanking exon h2 (with primers directed against exons v4 and h3). (**e**) CaMKIIβH is a minor splice also during mouse embryonal development (from embryonal day E10 to E18) and in mature mouse skeletal muscle and pancreas (two of the few non-brain tissues with any CaMKIIβ expression).

Surprisingly, our RT-PCR cloning approach (that was designed to detect exon v4 lacking variants) instead identified some unexpected new splice-variants. Two of seven clones lacking exon v1 had a slightly altered 3′-splice site on exon v2; in this new splice-variant (here termed βe-), an alanine is lost at the splice junction when compared to βe (Fig. 6a). A corresponding alternate 3′-splice site was never observed for full-length CaMKIIβ, and thus appears to be suppressed by inclusion of exon v1 in this major β variant.

More importantly, we identified two additional variants now termed βH and βeH, which both lack parts of hub domain exon h2 (Fig. 6b). While this deletion was not selected for, the same deletion was found in each of the two separate RT-PCR/restriction cloning approaches, with βH identified in cloning after BamHI digest and βeH identified in the cloning after SacII digest (and thus additionally lacking exon v1, similar to βe). The new βH variants are generated by usage of a splice site that is within exon h2. Importantly, the skipping of the first 78 nt of exon h2 in βH maintains the reading frame and the alternate 3′-splice site follows the general consensus sequence yncag/[A or G][G or T] (Fig. 6b). An identical splice consensus sequence is found in CaMKIIβ from rat, mouse, human, and zebrafish (Fig. 6c). Indeed, for human, a corresponding truncation of CaMKIIβ exon h2 is annotated in Ensembl (exon ENSE00001674946, in transcript ENST00000347193). A similar alternate splice consensus sequence is found also in exon h2 of rat CaMKIIδ, but not α or γ (Fig. 6c). However, RNA Seq in the rat hippocampal CA1 region detected an alternatively spliced exon h2 only in the CaMKIIβ isoform (in ~2% of all β transcripts), but in none of the other isoforms (Fig. 6c). However, the number of RNA reads for the δ isoform (for which potential alternative h2 splicing was also predicted) was dramatically lower than the reads for the β isoform (only ~200 reads for the δ isoform, i.e. ~2% of the reads for the β isoform); by contrast, the reads for the α and γ isoforms (for which no alternative h2 splicing was predicted) were ~190% and ~13% of the β isoform, respectively.

RT-PCR analysis of mouse brain with primers flanking exon h2 readily revealed expression of βH as a minor β splice-variant in all brain regions analyzed (Fig. 6d); identity of the shorter minor band with the expected length for βH was verified by sequence analysis from the whole brain RNA after a second round of PCR. In all brain regions analyzed, expression of CaMKIIβH was detectable, however, in each region only as a minor variant (Fig. 6d). Similarly, CaMKIIβH was found to be a minor variant also during prenatal development and in other tissues that express the CaMKIIβ gene (pancreas and skeletal muscle; Fig. 6e).

### The βH and βeH variants show reduced nuclear exclusion and F-actin localization

**Figure 7:**
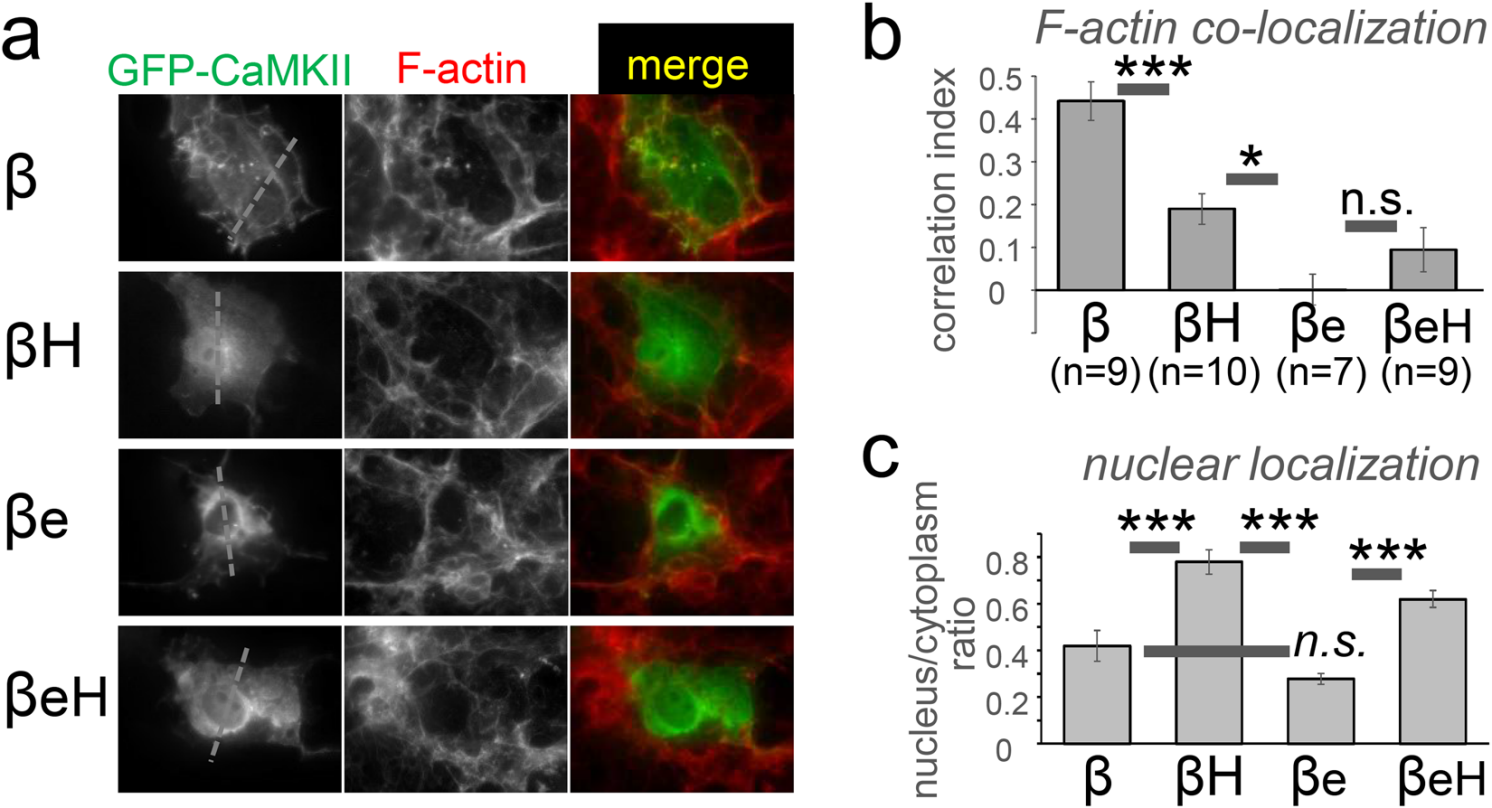
The partial hub domain deletion in the CaMKIIβH and βeH variants reduces nuclear exclusion and F-actin hub, indicating impaired holoenzyme formation. (**a**) Cos-7 cells expression various GFP-CaMKII constructs were fixed and F-actin was stained by Texas red-labelled phalloidin. (**b**) Quantification of nuclear localization revealed that the βH and βeH variants are significantly less excluded from the nucleus than the β and βe variants. (**c**) Quantification of co-localization with F-actin revealed significantly reduced localization of βH compared to β. Co-localization of F-actin binding-impaired βe variant was even lower, as expected, and not further reduced for the βeH variant.

The 12meric CaMKII holoenzymes are largely excluded from the nucleus, due to their size (>600 kDa). As the deletion of hub domain sequences in the new CaMKII splice-variant βH and βeH may impair holoenzyme formation, we first tested if they may show increased nuclear localization compared to the full-length CaMKIIβ. For this purpose, GFP-fusion proteins of the different CaMKIIβ splice-variants were expressed for two days in Cos-7 cells and then fixed (Fig. 7a). Quantification of the nucleus to cytoplasm ratio showed the same degree of nuclear exclusion for full-length CaMKIIβ and the βe variant; however, significantly less nuclear exclusion was seen for both βH and βeH variants (Fig. 7b), consistent with impaired holoenzyme formation.

CaMKIIβ can bind and bundle F-actin filaments^21,22^, and at least the latter property is thought to require the holoenzyme structure. Thus, we tested the CaMKIIβ variants for co-localization with F-actin, which was visualized in the fixed Cos-7 cells by staining with TexasRed-phalloidin (see Fig. 7a). Quantification showed significant colocalization of CaMKIIβ with F-actin, which was significantly reduced for the βH splice-variant (Fig. 7c). The βe variant has been shown previously shown to lack F-actin binding^21^; consistent with this previous observation, F-actin co-localization for βe was even lower and not further reduced for βeH (Fig. 7c). Together, these data indicate that the partial hub domain deletion in the splice-variants βH and βeH impairs the formation of normal holoenzymes.

### The βH and βeH variants can form heteromers with the full-length CaMKIIα hub domain

**Figure 8:**
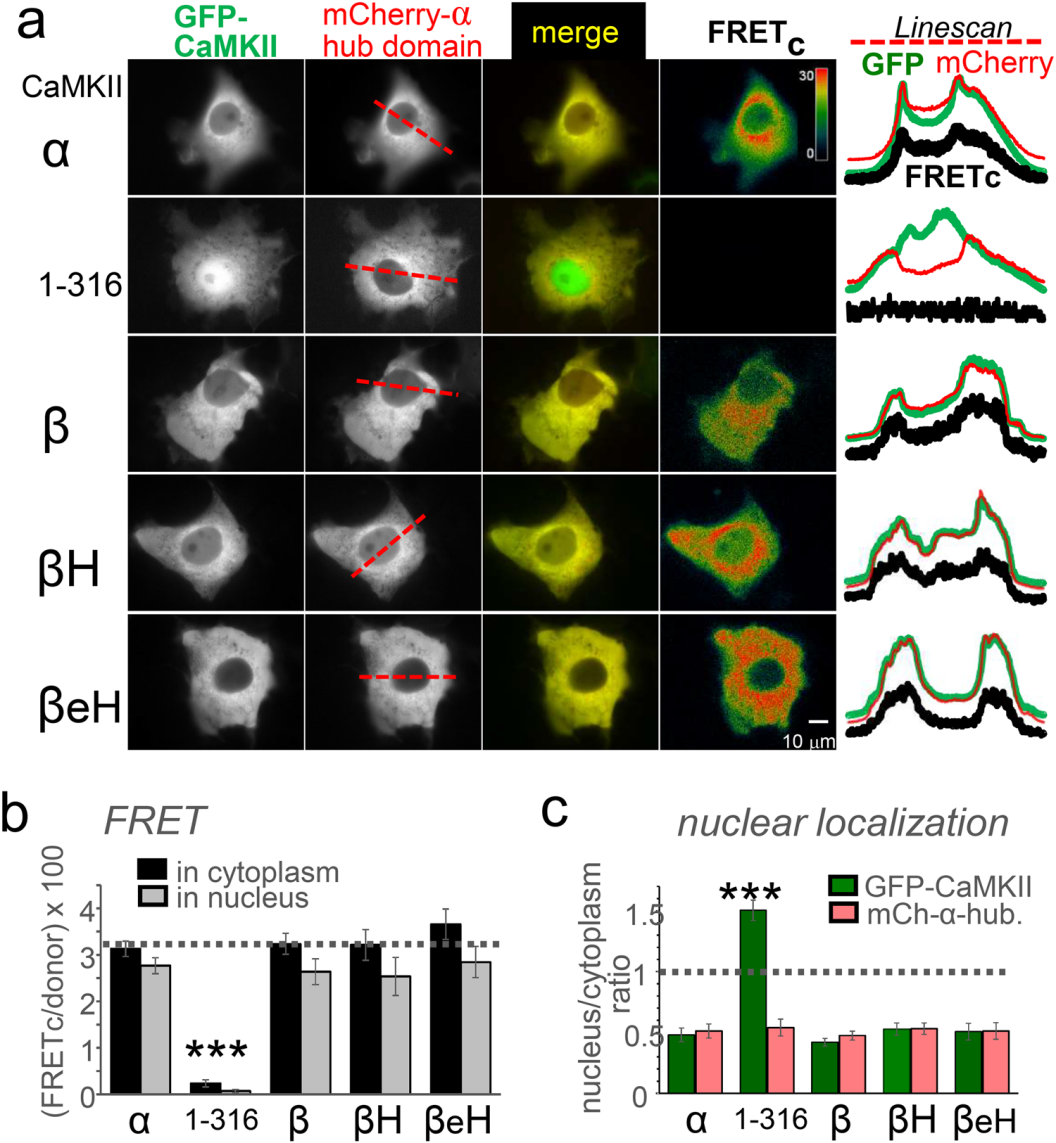
The CaMKIIβH and βeH variants can still co-assemble with the full-length CaMKIIα hub domain, as indicated by FRET assays and nuclear exclusion. (**a**) FRET-imaging of Cos-7 cells co-expressing an mCherry-labelled CaMKIIα hub domain with various GFP-CaMKII constructs; the shown line-scans were taken as indicated in the mCherry images. Robust corrected FRET (FRET_C_) was observed for all constructs, except for the truncated negative control 1–316, which completely lacks the association domain. (**b**) Quantification of the FRET signals in the cytoplasm and the nucleus. No significant differences were detected, expect for the truncated 1–316 negative control. (**c**) Quantification of nuclear localization revealed that co-expression with the CaMKIIα hub domain prevented any increase in nuclear localization for the βH and βeH variants. The only construct with increased nuclear localization was the truncated monomeric 1–316.

CaMKII holoenzyme formation can be probed within cells with a FRET assay^14^. A strong FRET signal is observed when full-length GFP-CaMKIIα is co-expressed with mCherry-labelled CaMKIIα hub domain in Cos-7 cells, and this FRET signal is essentially completely abolished for a GFP-CaMKIIα 1–316 construct that lacks the hub domain (Fig. 8a). A similar strong FRET signal was observed for full-length GFP-CaMKIIβ, and, somewhat surprisingly, for the βH and βeH variants (Fig. 8a,b). Thus, the βH and βeH variants both appear to be able to form heteromers with the full-length hub domain of CaMKIIα. Indeed, while individually expressed GFP-CaMKIIβH or βeH showed significantly more localization to the nucleus compared to GFP-CaMKIIβ (see Fig. 7b), co-expression with the mCherry-labelled CaMKIIα hub domain caused nuclear exclusion of both the βH and βeH variant (Fig. 8c). Together, these results indicate that the hub domain deletion in βH and βeH impairs formation of homomers, but still allows heteromeric interactions with the full-length hub domain of CaMKIIα.

### Optogenetic evaluation of CaMKII interaction within cells

**Figure 9:**
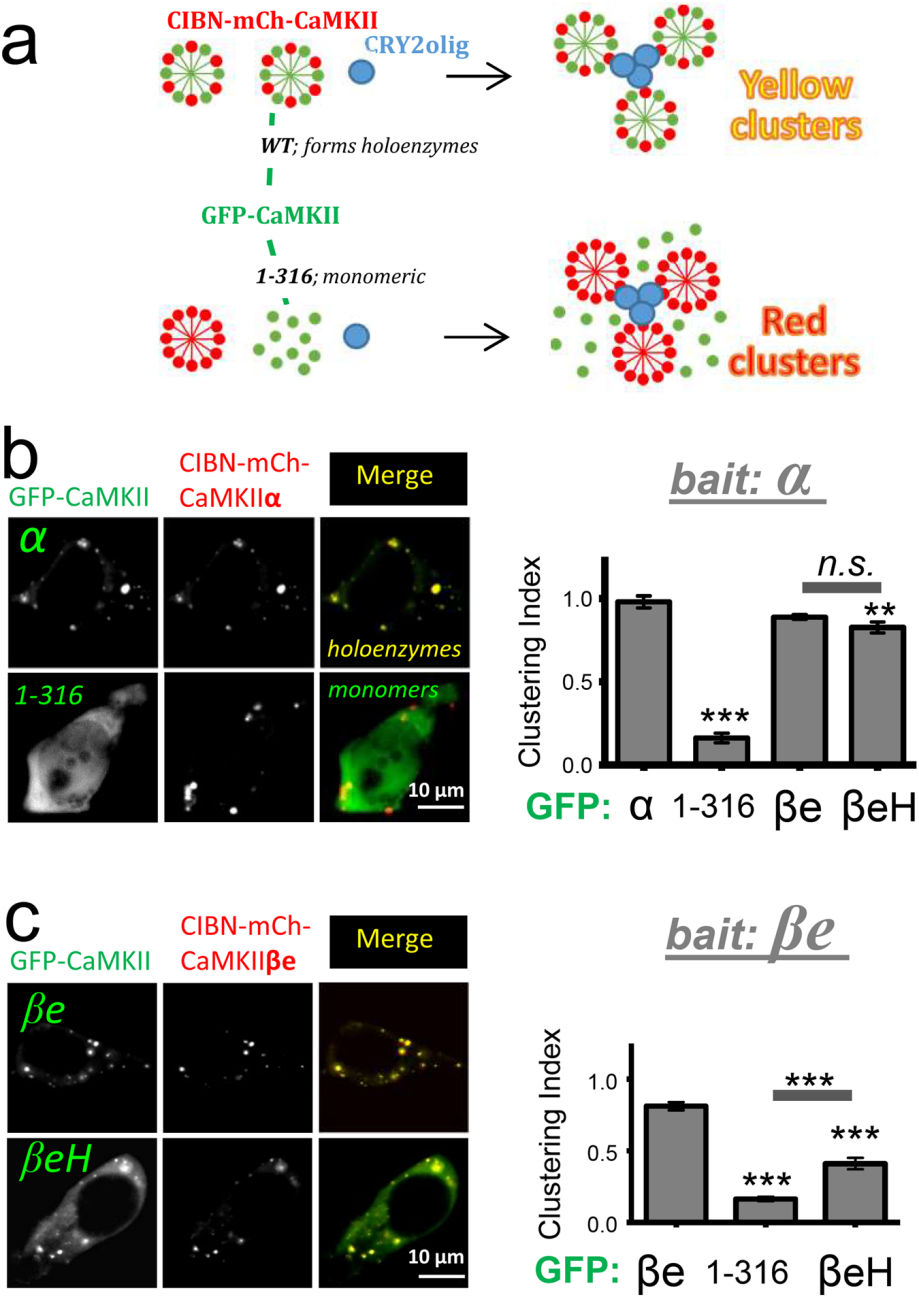
LINC analysis reveals impaired binding of the βH hub domain to CaMKIIβ but not α. (**a**) Schematic illustration of the LINC assay. Yellow clusters indicate formation of holoenzymes containing WT GFP-CaMKIIα and CIBN-mCh-CaMKII bait. Red clusters indicate inability of monomeric 1–316 truncation to form holoenzymes with CIBN-mCh-CaMKII bait. (**b**) Representative images of HEK cells expressing full-length GFP-CaMKIIα or the 1–316 truncation that completely lacks the hub domain along with CIBN-mCh-CaMKIIα and CRY2olig. Quantification of LINC imaging reveals co-clustering with CIBN-mCh-CaMKIIα as bait was seen for CaMKIIα and the CaMKIIβe and βeH splice-variant mutants but not for the monomeric 1–316 negative control. Data represent mean ± SEM. n = 5–9 cells. **p < 0.01, ***p < 0.001. Scale bars, 10 µm. (**c**) Representative images of HEK cells expressing GFP-CaMKIIβe or βeH as well as CIBN-mCh-CaMKIIα and CRY2olig. Quantification of LINC imaging reveals co-clustering with CIBN-mCh-CaMKIIβe as bait was seen for CaMKIIβe but co-clustering was significantly reduced with both the hub-domain lacking CaMKIIβeH variant and the 1–316 negative control. Data represent mean ± SEM. n = 14–28 cells.***p < 0.001. Scale bars, 10 µm.

A more direct assessment CaMKII binding interaction within cells was done by a novel optogenetic method that we recently developed: light-induced co-clustering (LINC)^36^. The LINC assay is based on the light-induced binding of CRY2olig (an optimized version of the *Arabidopsis* photoreceptor cryptochrome 2, CRY2)^36^ to CIBN (a truncated version of cryptochrome-interacting basic-helix-loop-helix protein 1, CIB1)^37,38^. When co-expressed with CRY2olig, a CIBN-fused and mCherry-labelled CaMKII (CIBN-mCh-CaMKII) forms clusters after stimulation with blue light^36,39^. When GFP-CaMKII is co-expressed as third protein, it should co-cluster with CIBN-mCh-CaMKII, based on formation of CaMKII holoenzymes via their hub domains. By contrast, GFP-CaMKII variants or mutants that are impaired for holoenzyme formation (such as the CaMKIIα 1–316 mutant that lacks the entire hub domain) should fail to co-cluster with CIBN-nCh-CaMKII (Fig. 9a). Indeed, co-clustering with the CIBN-mCh-CaMKIIα was observed in HEK cells for GFP-CaMKIIα wild type, but not for the monomeric GFP-CaMKIIα 1–316 (Fig. 9b), as expected. For testing co-clustering mediated by the CaMKIIβ hub domains, we utilized the βe splice-variants that reduce F-actin binding, in order to minimize any possible interference of the F-actin interaction with the formation of clusters. Extensive co-clustering of both GFP-CaMKIIβe and βeH was observed when CIBN-mCh-CaMKIIα was used as bait (Fig. 9b). While co-clustering of βeH was slightly but statistically significantly reduced compared to α, there was no significant difference between βeH and βe (or between βe and α; Fig. 9b). However, when the bait was instead CIBN-mCh-CaMKIIβe, the co-clustering seen for GFP-CaMKIIβe was reduced by half for βeH (Fig. 9c). This provides direct evidence that the exon h2 deletion in βH and βeH significantly impairs binding to the full-length hub domain of CaMKIIβ, but affects binding to CaMKIIα only mildly at best.

## Discussion

Together, the results of this study provide (i) reference for the distribution of CaMKIIα and β splice-variant among different brain regions and (ii) the first evidence for isoform-specific differences in the hub domain interactions that mediate holoenzyme formation. Most notable about the splice-variant distribution was the widespread expression of the nuclear CaMKIIαB, a splice-variant previously described in rat midbrain^16^. The αB variant was detected in all brain regions analyzed, and was found to be even the dominant α variant in hypothalamus and brainstem (in both pons and medulla). The significance of the αB splice-variant lies in the fact that it contains the insertion of the exon v3_N_, which generates a functional nuclear localization signal^16,17,28^ that is similar to the originally described nuclear localization signal of the SV40 large-T antigen^40^. Indeed, a variant of CaMKIIγ with a similar nuclear localization signal has been prominently described to function in Ca^2+^-dependent excitation/transcription coupling in neurons^28^. In this case, calcineurin was required to dephosphorylate sites near the nuclear localization signal, which in turn allowed translocation of CaMKIIγ from the cytosol to the nucleus^28^. The CaMKIIαB variant contains homologous sites, and their phosphorylation also blocks nuclear localization^41,42^, suggesting the possibility for a similar regulation of the localization of αB in neurons. Notably, while αB is only a minor α splice-variant in most brain regions, the overall expression of the CaMKIIα isoform in brain is much higher compared to the γ isoform^3,4^. Indeed, analysis of our RNA Seq indicated that the rat hippocampal CA1 region contains slightly (10–20%) more exon v3_N_-containing α than γ transcripts. Additionally, while a nuclear function of CaMKIIγ has been suggested also in cortical neurons, it has been studied mainly in neurons cultured from the superior cervical ganglion^28^.

For mammalian CaMKIIβ, no variants have been described that contain the nuclear localization signal generated by inclusion of exon v3_N_. In fact, while rat, mouse, and human α, γ and δ isoforms all have variants that include exon v3_N_, the β isoform gene from these species were found to completely lack exon v3_N_. However, β isoform cDNAs from various fish species was found to contain exon v3_N_, suggesting that this exon was eliminated from the CaMKIIβ gene later during vertebrate evolution. While the reason for deletion of the nuclear localization signal in CaMKIIβ is unclear, it could be related to the β specialization to provide high-affinity F-actin binding in the cytoplasm^20–24^. While the mammalian CaMKIIβ isoform lacks exon v3_N_, it contains three exons that are lacking in the α isoform, exon v1, v4, and v5 (see Fig. 1). These exons are also present in the CaMKIIγ and δ genes, where they are all subject to alternative splicing^31^ (note that the terminology for the variable region in this reference differs slightly, as they chose to sub-divide variable exon v4 into two segments). By contrast, essentially all CaMKIIβ transcripts in rat brain were here found to contain exon v4 (similar to exons v2 and v6, which appear to be present in almost all splice variants of all CaMKII isoforms). The significance of such obligatory inclusion of variable exon v4 is not entirely clear. However, the length of the variable linker is known to effect parameters such as Ca^2+^/CaM-sensitivity, the frequency-dependent response to Ca^2+^-oscillation frequencies, and substrate selectivity^15,18,29,43^. Nonetheless, while by far the majority of CaMKIIβ transcripts in the mature brain corresponds to the full-length β variant, the lack of exon v4-lacking transcripts is somewhat striking, as transcripts lacking exon v1 or v5 (corresponding to βe or β’) were readily detectable. While the effect of exon v4 deletion on F-actin binding of CaMKIIβ is not known, this binding is disrupted by deletion of exon v1 but not v5^21^ (note that the numbering of variable exons in this reference differs from the terminology used here, as this reference did not account for exon v3_N_).

Alternative splicing of the CaMKII variable linker exons is common, and has been described to affect both CaMKII regulation and localization. By contrast, alternative splicing of exons coding for the other domains is uncommon. While there is an alternative product of the CaMKIIα gene that completely lacks the kinase domain, this lack is due to use of an alternative promoter, not alternative splicing^44^. This alternate gene product is termed αKAP and contains the complete association domain^44^; it thought to function in targeting various CaMKII isoforms to the sarcoplasmic reticulum membrane in skeletal muscle^6,44,45^ and in the postsynaptic clustering of acetylcholine receptors at the neuromuscular junction^46^. By contrast, to our knowledge, the βH and βeH variants described here are the first known mammalian CaMKII splice-variants that lack part of the hub domain and show impaired formation of the 12meric CaMKII holoenzymes. While CaMKIIβ lacks any nuclear localization, the impaired holoenzyme formation of the βH and βeH variants could indirectly enhance nuclear localization: While the large CaMKII holoenzymes are excluded from the nucleus due to their size, the individual subunits are small enough to fall below the size exclusion limit. Indeed, the βH and βeH variants showed increased nuclear localization in Cos-7 cells. However, surprisingly, co-expression of the CaMKIIα hub domain prevented such increase nuclear localization. This indicated that the truncated hub domain of βH and βeH is incapable of forming homomeric holoenzymes, but is still able to form heteromeric interactions with full-length hub domain. Indeed, such heteromeric interactions of the βH hub domain with the full-length CaMKIIα hub domain were also found using our optogenetic binding assay. However, surprisingly, the interaction of the βH hub domain with the full-length CaMKIIβ hub domain was instead dramatically impaired. Thus, the interaction of βH with other hub domains did not depend on whether or not they also have a partial hub domain truncation, but instead on the type of isoform, i.e. α versus β. To our knowledge, this is the first observation of such isoform-specific differences in the hub domain interactions that mediate holoenzyme formation. The more robust interaction with α compared to β hub domains suggests that the α isoform may provide more or stronger interaction sites and therefore possibly tighter binding. Thus, the exchange of subunits within preformed holoenzymes may be easier for β versus α subunit. So far, such subunit exchange has been studies largely for the α subunit, and is thought to involve transition through 14meric holoenzymes^14,47–49^.

The widespread but very low-level expression of the novel βH variants that lack part of the hub domain leave their functional significance in cellular signaling unclear. However, their differential interactions provide first insights into isoform-specific differences in CaMKII holoenzyme formation. Holoenzymes are required for major regulatory aspects of CaMKII, such as the inter-subunit T286-autophosphorylation that enables frequency detection^50–52^ and is required for bi-directional long-term synaptic plasticity^9,11^. Thus, understanding the mechanisms that govern holoenzyme formation, maintenance, and turnover will be worthwhile goals for further studies.

## Methods

### Materials

All materials were obtained from Sigma, unless indicated otherwise. GFP-CaMKII constructs^20,21^ and CIBN-mCherry-CaMKII constructs^36^ were described previously, and were here further modified as indicated.

### Animals

All studies conformed to the requirements of the National Institutes of Health Guide for the Care and Use of Laboratory Animals and were approved by the Institutional Animal Care and Use subcommittee of the University of Colorado, Denver AMC. Timed-pregnant Sprague Dawley rats (Charles Rivers Labs, Wilmington, MA) gave birth in house. Dams were housed in micro-isolator cages with water and chow available ad libitum.

### RT-PCR expression analysis

RT-PCR was performed essentially as described^21^, using the RED Extract-N-Amp PCR ReadyMix (R-4775-1; Sigma) on commercially obtained cDNAs from mouse brain regions, tissues, and embryonic stages (Zyagen, San Diego, CA), using 34 amplification cycles. The CaMKIIα variable region was amplified using primers against exon r1/2 (AAGGGAGCCATCCTCACCACTATG) and exon h3 (GATGAAAGTCCAGGCCCTCCAC), with an annealing temperature of 65 °C; the CaMKII variable region was amplified with primers against exon r2 (CAGACAGGAGACTGTGGAATGTCTG) and exon h1 (GCCTCAAAGTCCCCATTGTTGA C), with an annealing temperature of 63 °C. For detection of the novel βH variant, CaMKIIβ exon h2 was flanked by primers against exon v4 (GCCTCAAACCACCGTTATCCATAACCC) and exon h3 (GCGATGCAGGCTGCATCCTCGC CGATG), and amplified using an annealing temperature of 63 °C. Note that the primers flanking the variable region were derived from the mouse sequence, while the exon a2 flanking primers where derived from the rat sequence (which is identical to mouse for the forward primer, but differs at three positions for the reverse primer). However, identity of the two bands as β and βH was confirmed by sequence analysis: The first-round PCR amplificate from whole brain cDNA was digested with TaqI (cutting in the exon h2 region that is missing in βH) and then used as template for a second round of PCR that again resulted in two bands (but now in more equal amounts); both bands were directly sequenced after gel extraction and purification.

### Western-blot analysis

Olfactory Bulb, hippocampus, hypothalamus, cerebellum and brain stem were dissected from 8–12 week old C57Bl/6 mice. The dissected brain tissue was first sonicated in a buffer containing 10 mM Tris pH 8, 1 mM EDTA, and 1% SDS and then boiled for 5 min. Total protein concentrations were determined using a Pierce BCA protein assay kit (Thermo Scientific). Western-blot analysis after protein transfer onto PVDF membrane was done essentially as described previously^12,53–55^, using antibodies specific for CaMKIIβ (CBβ1) or CaMKIIα (CBα2). Chemo-luminescence detection was done using Super signal west femto ECL reagent (Thermo Scientific) for 3 minutes; images were acquired on a Fluorchem SP imager (Alpha Innotech). The 10% polyacrylamide SDS gels were precast criterion gels (BioRad); other percentage gels were made in house.

### RNA sequencing

Hippocampal CA1 parasagittal slices were prepared as previously described^56^, from day 14 postnatal rats. Total RNA was extracted using a Trizol/Chloroform method. Tissue was homogenized in Trizol (1 ml per mg) and centrifuged for 3 min at 14000 rpm at 4 °C. Then, the supernatant and 250 μl of chloroform was added to Phaselock gel Heavy 2 ml (5Prime), and centrifuged for 5 min at 13000 rpm. An additional 250 μl of chloroform was added and centrifuged again 10 minutes. RNA was further purified from the top clear layer using the Qiagen RNeasy minElute protocol. Total RNA was stored at −80 °C, and then submitted to the University of Colorado Genomics and Microarray core for further processing and sequencing. 500 ng of total RNA was used to prepare the Illumina HiSeq libraries according to manufacturer’s instructions for the TruSeq stranded mRNA protocol. Briefly, mRNA was isolated from total RNA using polyA selection and primed for creation of double-stranded cDNA fragments which were subsequently amplified, size selected, and purified for cluster generation. RNA was sequenced in two rounds of 4 samples each, pooled over two lanes and sequenced using the Ilumina HiSeq. 2500 or HiSeq. 4000 platform, generating either 125 or 150 base, paired end reads. RNA sequencing data were aligned to the Ensembl Norway Rat reference genome 6.0 using SamTools. Genome aligned read data were loaded into IGV (Broad Institute), and chromosome locations of exons of interest were determined and used to generate Sashimi plots for splice analysis.

### RT-PCR/restriction strategy for detection of exon v4-lacking β transcripts

RT-RCR was performed on cDNA prepared from adult rat brain as described previously^21^, using 35 cycles with high-fidelity “Platinum” Taq polymerase (Invitrogen). First round primers were directed against CaMKIIβ nt 69–87 (GGCTTTCTCTGTGGTCCG) and 1717–1737 (AGGGGAGGGGACGAGGCA); the nested second round primers were directed against nt 408–427 (CCTCAAGCCTGAAAACCT) and 1648–1667 (ACAACTCTGGTCCGG), with numbering of nucleotides based on reference^57^. Prior to the nested second round PCR, primers were removed from the first round PCR products using spin columns (Quiagen) and the PCR products were digested with either SacII or BamHI. Products from the second round were again digested, and the restriction resistant fragments were gel purified and then directly cloned into pXcmI (a pUC19 based TA-cloning vector)^58^ for further analysis.

### Imaging of CaMKII subcellular localization in Cos-7 cells

All microscopic imaging was performed using a 100 × 1.4NA objective on a Zeiss Axiovert 200 M (Carl Zeiss, Thornwood, NY), controlled by SlideBook software (Intelligent Imaging Innovations, Denver, CO). Cos-7 cells were transfected with GFP-CaMKII expression vectors, using the calcium phosphate method. For determining co-localization of GFP-CaMKII with F-actin, cells were fixed in 4% paraformaldehyde two days after transfection, permeabilized with 0.1% Triton-X100, stained for F-actin with 165 nM Texas Red-phalloidin (Invitrogen). Images were acquired as 2.2 µm z-stack (with 0.2 µm step size). Analysis of fixed cell images was performed using maximum z projection images after nearest neighbor deconvolution in Slidebook, followed by Pearson’s correlation to determine the co-localization of GFP-CaMKII and F-actin, essentially as described previously^21^.

Analysis of nuclear localization was done with the same software, either on the same images of the fixed cells or on the images of live cells taken for the FRET analysis. For each fixed or live cell image, three masks were made to assess the mean intensity in the nucleus, cytoplasm, and background. All cytoplasm masks were comprised of a region immediately surrounding the nucleus in order to avoid cell edges.

### CaMKII subunit interaction assessment by FRET imaging

Cos-7 cells were transfected with mCherry and GFP expression vectors (4:1 ratio) by the calcium phosphate method and imaged 24 h later. Acquisition and analysis of FRET images utilized SlideBook software (Intelligent Imaging Innovations, Denver, CO), and was performed as described previously in detail^14,59^. Briefly, corrected FRET (FRET_C_) was divided by the intensity of the FRET donor in order to normalize for expression levels; this method has been validated for situations when donor is in access over the acceptor^14,59^, as was the case here.

### Immunohistochemistry

For tissue isolation an preparation, mice were transcardially perfused under isoflurane anesthesia with phosphate-buffered saline for five minutes, followed by five minutes of fixation with 4% paraformaldehyde. Their brains were removed, allowed to post-fix in 4% PFA for 24 hours, and embedded in paraffin, as previously described^60,61^. Coronal sections were cut in 6 µm thickness, and every sixth section was mounted in series onto slides for further processing. The interval between serial slide groups was 100 µm. For CaMKII staining, slides were washed with PBS, blocked for 1 hour (5% normal donkey serum with 0.3% Triton X-100), then incubated for 24 hr with primary antibody at 4oC. Mouse anti-CaMKII (CBα2) was diluted in blocking solution at 1:1000 concentration and rabbit anti-NeuN (Millipore) was diluted in blocking solution at 1:500 concentration. After washing with PBS, sections were incubated with appropriate secondary antibodies, Alexa Fluor 488, 594 (Jackson Immuno or Abcam) for 1 hr at room temperature. Imaging and analysis used the same microscope setup and software as for the Cos-7 cell imaging; however, the objective used was a Zeiss 40×.

### Optogenetic assessment of CaMKII subunit interaction

HEK293 cells were transiently transfected by the calcium phosphate method as previously described^6,21^, using a 1:1:1 ratio of GFP-labeled CaMKII (α, 1–316, βe, or βeH), CIBN-mCh-labeled bait CaMKII (α or βe), and unlabeled CRY2olig^36^. 24 h after transfection, transfected cells were identified using blue light to activate CIBN-CRY2olig clustering, and images were acquired 1 minute after the initial blue-light exposure.

Live imaging of cells was carried out at 32 °C in HEPES-buffered imaging solution containing (in mM): 130 NaCl, 5 KCl, 10 HEPES (pH 7.4), 20 glucose, 2 CaCl_2_, and 1 MgCl_2_. Focal plane z stacks (0.3-µmsteps; over 1.8–2.4 µm) were acquired and deconvolved to reduce out-of-focus light. 2D maximum intensity projection images were then generated and analyzed by an experimenter blinded to GFP-CaMKII isoform and “bait” CIBN-mCh-CaMKII using Slidebook 6.0 software.

Clustering of GFP-CaMKII isoforms with CIBN-bait CaMKII was quantified via clustering index (GFP/mCh ratio in clusters vs. non-clustered regions). Clusters were identified using a threshold mask of the CIBN-mCh fluorescence intensity (mean +2 st dev of whole cell mCh intensity) for each cell. Non-clustered regions were characterized by subtracting the mean fluorescence intensity of the clusters from the intensity of the whole cell. Mean GFP and mCh background intensity was subtracted from each cell prior to quantifying the clustering index.

### Statistical analysis

All quantifications are shown as mean ± SEM. Data were analyzed by ANOVA followed by posthoc analysis with Neuman-Keuls test. Statistical significance is indicated, including by *p < 0.05; **p < 0.01; ***p < 0.001. The datasets generated during and/or analysed during the current study are available from the corresponding author on reasonable request.
